# Assessment of Floral Nectar and Amino Acid Yield in Eight Landscape Trees for Enhanced Pollinator Food Resources in Urban Forests

**DOI:** 10.3390/plants14131924

**Published:** 2025-06-23

**Authors:** Sung-Joon Na, Ji-Min Park, Hae-Yun Kwon, Young-Ki Kim

**Affiliations:** 1 Special Forest Resources Division, National Institute of Forest Science, Suwon 16631, Republic of Korea; 2 Department of Forest Resources, Sunchon National University, Suncheon 57922, Republic of Korea

**Keywords:** urban tree, floral nectar, food availability, sugar content, amino acid composition

## Abstract

Urban environments pose challenges for pollinators due to habitat loss and limited floral resources. However, green infrastructure, particularly street and ornamental trees, can play a critical role in supporting urban pollinator communities. In this study, we evaluated nectar volume, sugar content, and amino acid composition across eight urban tree species commonly planted in South Korea. Using standardized productivity metrics at the flower, tree, and hectare scales, we compared their nutritional contributions. Our results revealed substantial interspecific differences in nectar quantity and composition. *Tilia amurensis*, *Heptacodium miconioides*, *Aesculus turbinata*, and Wisteria floribunda exhibited high nectar yields or amino acid productivity, whereas species such as *Cornus kousa*, though lower in nutritional yield, may offer complementary value due to their distinct flowering periods or other phenological traits. These findings underscore the importance of selecting tree species not only for aesthetic value but also for ecological function, providing an evidence-based approach to pollinator-friendly urban biodiversity planning and landscape management.

## 1. Introduction

Plant–pollinator interactions are essential for the survival of both entities [1,2]. Pollinators depend on the nectar and/or pollen sourced from plants as nutritional resources vital for their growth, development, and immune system maintenance—both at the individual and colony levels [3,4]. In recent years, concerns regarding potential declines in certain pollinator species in some areas have intensified [5,6,7]. This decline is believed to arise from stressors such as habitat loss, parasites, diseases, pesticides, scarcity of flowers, and climate change, which may act individually or collectively to exacerbate the situation [8]. The reduction in the abundance and diversity of pollinators jeopardizes ecosystem sustainability [9] and can hinder agricultural crop production, posing risks to food security [10]. Consequently, the significance of pollinators is being increasingly acknowledged.

Urban areas are typically regarded as less suitable habitats for pollinators owing to various factors, including habitat loss, fragmentation, and environmental pollution [11]. Deforestation and soil sealing, accompanying urban expansion, significantly contribute to the reduction of plant resources, leading to a shortage of both food and nesting sites for pollinators [12]. Urbanization-induced decline in pollinators has been observed not only in wild pollinator populations [13,14] but also in managed honeybee colonies [15,16]. While many studies report that urbanization contributes to pollinator declines through habitat fragmentation and floral resource loss, recent global syntheses reveal more nuanced patterns. For example, urban land use has been associated with reduced pollinator abundance in some contexts [17], yet certain urban areas can act as biodiversity hotspots for specific pollinator taxa, such as bees [18]. This variability underscores the importance of evaluating floral resource quality and availability within urban environments.

Urban green spaces are increasingly recognized as beneficial for the recovery of pollinator populations [19]. In recent decades, many cities around the world have planted a more diverse range of plant species as part of urban greening initiatives and in response to growing awareness of the importance of biodiversity within urban ecosystems. This diversification has increased overall plant richness [20], which in turn has contributed to greater floral resource availability and extended phenological continuity—both of which are key drivers of pollinator diversity [21,22]. In particular, urban environments may be especially favorable for pollinators due to reduced pesticide exposure [23] and the intentional creation of complex, heterogeneous plant assemblages for landscaping purposes [18,24]. The high pollinator diversity and abundance observed in such urban green spaces present valuable opportunities to develop pollinator-friendly urban environments [25,26,27,28,29].

Urban forests, formed by street and ornamental trees, not only contribute to human well-being and mental health [30,31] but also serve as critical food resources for pollinators [32,33,34,35]. Specifically, woody plants are recognized as a cost-efficient and long-term approach for supporting pollinator populations [36,37,38,39]. This is attributed to the capability of woody plants to provide abundant floral nectar and pollen [36,40]. Therefore, careful consideration is required in selecting tree species for urban planting to ensure harmonious coexistence between humans and ecosystems. Nevertheless, the selection of street trees currently relies predominantly on human-centered values such as aesthetic appeal, air pollution mitigation, pest sensitivity, and maintenance costs [41,42]. The aspect of providing food resources for pollinators in urban areas has not been adequately addressed [43]. Therefore, to effectively manage floral resources in urban areas, it is imperative to gather extensive knowledge regarding the quantity and quality of floral rewards provided by urban trees [44].

Floral nectar serves as a high-energy resource for most pollinators and plays a central role in their foraging behavior and energy metabolism [45]. While both nectar and pollen contribute to pollinator nutrition, nectar is particularly critical for adult insects such as bees and butterflies, many of which rely on it as their primary source of carbohydrates during active foraging and reproduction [46,47]. Therefore, assessing nectar availability is essential for understanding the ecological support urban plants can offer to pollinators. This nectar-focused framework allows us to evaluate the quality and quantity of floral resources that urban trees can provide to support and sustain pollinator populations. As this study focuses on nectar-based food provisioning, the term ‘pollinators’ primarily refers to insect taxa—such as honeybees and butterflies—that rely heavily on floral nectar as a key nutritional source.

While nectar is primarily known as a carbohydrate source, it also contains a variety of amino acids that play a crucial role in determining nectar quality [48,49] and may influence pollinator foraging behavior and preferences [50]. However, pollen is generally recognized as the principal source of amino acids for most pollinators [51]. Therefore, although the quantitative contribution of nectar amino acids may be limited, their functional roles in pollination dynamics remain of ecological interest. Honeybees consuming a balanced intake of amino acids demonstrate improvements in lifespan and fecundity [52], along with positive effects on memory and learning capabilities [53,54]. Nevertheless, research focusing on amino acid availability remains limited [55,56]. The concentration and composition of sugars and amino acids in floral nectar vary significantly among plant species [57,58,59,60]. Therefore, understanding both the qualitative and quantitative aspects of nectar across different plant species is essential for gaining a deeper insight into food availability for pollinators [61,62,63,64]. Although numerous studies have investigated sugar and amino acid composition in floral nectar [38,63], comparative analyses integrating both traits across multiple urban tree species and productivity scales remain limited.

In this study, we analyzed the availability and characteristics of floral nectar and amino acids from eight tree species commonly employed as street and landscaping trees to determine the foundational food resources for pollinators in urban areas. Although *Wisteria floribunda* is technically a woody vine and *Heptacodium miconioides* is a shrub, both are functionally planted and managed as small trees in urban landscaping. For simplicity, we refer to all eight species as ‘trees’ in this study. The selected tree species represent a policy-relevant and ecologically practical subset of urban trees in South Korea. They are widely planted in cities, officially recognized as nectar-producing plants under national greening regulations [65,66], and collectively span a broad blooming period from early spring to early fall, ensuring continuous floral resource availability for pollinators.

By comparing nectar productivity and chemical quality at multiple biological and spatial scales (per flower, per tree, per hectare), our study aims to identify species that should be prioritized to support richer and more diverse food resources for urban pollinators. The findings may inform ecologically functional plant selection in urban greening strategies and contribute to more standardized approaches in interspecific comparisons.

## 2. Materials and Methods

### 2.1. Study Site and Tree Species

The trees under investigation were planted for landscape purposes at the Division of Forest Biological Resources, National Institute of Forest Science, Suwon, South Korea (37°15′55″ N and 126°57′29.9″ E, elevation: 44 m). This region exhibits the characteristics of a continental climate, with an average annual temperature of 12.5 °C. The coldest month is January, with an average temperature of −2.1 °C, and the hottest month is August, with an average temperature of 26.0 °C. The average annual precipitation is 1320.3 mm, and the average annual relative humidity is 68.3%. The site is classified as Dwa (hot-summer humid continental climate with dry winter) under the Köppen–Geiger climate classification system.

**Figure 1:**
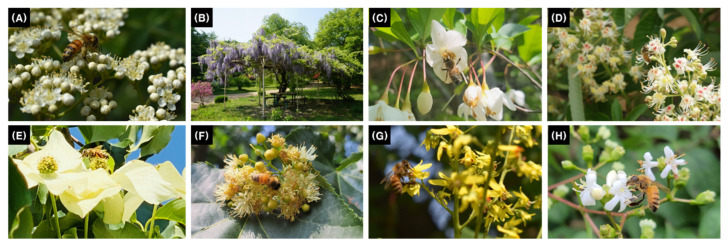
Flower morphology and floral nectar foraging by honeybees in eight urban tree species: (**A**) *Sorbus commixta* Hedl., (**B**) *Wisteria floribunda* (Willd.) DC., (**C**) *Styrax japonicus* Siebold & Zucc., (**D**) *Aesculus turbinata* Blume, (**E**) *Cornus kousa* Bürger ex Hance, (**F**) *Tilia amurensis* Rupr., (**G**) *Koelreuteria paniculata* Laxm., (**H**) *Heptacodium miconioides* Rehder.

The study species comprised eight tree species commonly planted in parks and as street and ornamental trees in the urban areas of South Korea (Figure 1). They were thriving without pest damage and competition from surrounding trees. Three to ten trees per species were used in the experiment. The tree species used in this study are included in the list of 618 honey plants in Korea, as documented by Jang (2008) [65], and have long been utilized as honey plants. Notably, *Sorbus commixta* Hedl. (Rosaceae), *Styrax japonicus* Siebold & Zucc. (Styracaceae), *Aesculus turbinata* Blume (Sapindaceae), *Tilia amurensis* Rupr. (Malvaceae), and *Koelreuteria paniculata* Laxm. (Sapindaceae) are designated as honey plants under Korea’s “Act on the Promotion and Support of the Beekeeping Industry” [66] and are recommended for the establishment of honey plant complexes. The Act, enacted in 2020, aims to protect and promote honey plants, including 25 species of woody plants and 15 species of herbaceous plants. *Wisteria floribunda* (Willd.) DC (Fabaceae) and *Cornus kousa* Bürger ex. Hance (Cornaceae) are recognized as excellent honey plants by Korean beekeepers. *Heptacodium miconioides* Rehder (Caprifoliaceae) is a newly identified honey plant in this study.

### 2.2. Period and Number of Flowering

We recorded the start and duration of blooming in 2022. Blooming was considered to have started when more than 5% of the total flower buds had bloomed. Full bloom was recorded when the cumulative blooming rate reached 45–55%, and termination of blooming was recorded when more than 80% of the flowers had bloomed and withered [35].

The total flower number per tree was determined by first recording the total number of inflorescences and then multiplying this value by the flower number obtained from sampling 30 inflorescences per tree [33]. The flower number per inflorescence was measured when the cumulative flowering rate was below 40%, as this stage allows for easier counting when the flowers are still in bud and helps minimize errors caused by flower drop. Both bloomed and unbloomed flowers (i.e., in the bud stage) were included in the total flower number.

In addition to flowering phenology, we measured the height and crown width of each tree to evaluate growth traits. These data were used to calculate tree density (trees/ha), which we estimated by dividing 10,000 m^2^ by the canopy area (square of crown width) of each tree, assuming non-overlapping crowns. When evaluating the value of available food resources for pollinators, the most important factor is the total amount of resources available per unit area of each individual woody plant [35,67,68]. However, accurately assessing the available food resources in trees is generally challenging due to several variables, such as the age of the tree, crown shape, and flower abundance [68]. Moreover, finding survey trees that meet the same conditions (either the same age or trees with the same crown area, even if they are of different ages) may be practically impossible. Therefore, in this study, although street and ornamental trees are not planted on a per-hectare basis, we standardized our data to allow for quantitative comparisons between species. We calculated the number of trees per hectare based on the canopy area (square of crown width) of the survey tree, which determines the space needed between trees [69].

### 2.3. Measure of Floral Nectar Volumes

Floral nectar volume measurements were conducted at the full bloom phase for each tree. All previously bloomed flowers were removed at 4:00 p.m., and unopened buds were enclosed in pollination bags with a pore size of 0.3 mm to prevent nectar loss and insect visitation [70]. To minimize spatial bias, flowers were selected from inflorescences located in different parts of the crown (upper, middle, and lower branches) and from different orientations (north, south, east, and west), depending on accessibility.

Each bag covered a single inflorescence. Nectar was collected from flowers that opened after bagging and completed their full lifespan. Although floral nectar is secreted throughout the flower’s open period, we used cumulative nectar volume as a proxy for per-flower yield. This approach was supported by our prior study [71], which showed no significant difference between two-day cumulative collection and the sum of daily extractions using capillary tubes. All eight tree species examined had flowers with a consistent lifespan of two days.

Nectar sampling was performed on 3 to 10 individual trees per species, depending on tree availability. We collected at least 100 flowers on the second day after bagging them. Afterward, we carefully removed unnecessary parts (such as petals and peduncles) to the extent that nectar loss was minimized, and we placed the flowers in a 50-mL tube equipped with nylon mesh. These tubes were centrifuged at 4000 rpm for 4 min [72,73]. The floral nectar collected in the tubes was quantified using a 50–100 μL syringe (Hamilton Co., Reno, NV, USA), and to prevent microbial-mediated decomposition of the floral nectar, a tenfold dilution of 80% ethanol (*v*/*v*) was added [74,75]. Finally, to remove foreign matter and pollen, the floral nectar was further purified using a 0.45-μm centrifugal filter, followed by storage at –20 °C until analysis of sugar and amino acid contents.

### 2.4. Sugar Content and Composition

The analysis of free sugar content was performed using an HPLC system (Dionex Ultimate 3000; Dionex, Sunnyvale, CA, USA). The mobile phase was deionized water at a flow rate of 0.5 mL/min, and the column oven was maintained at 80 °C. Detection was achieved using a Shodex Ri-101 (Showa Denko America, Inc., New York, NY, USA) coupled with an Aminex 87P column (300 × 7.8 mm, Bio-Rad Laboratories, Hercules, CA, USA). Free sugars were quantified using high-purity standards (99.5%) of sucrose, glucose, and fructose (Sigma-Aldrich, St. Louis, MO, USA). The sugars identified in each type were used to calculate the sucrose/hexose (glucose + fructose) and glucose/fructose ratios [50].

In addition, Pearson’s correlation analyses were performed to assess the relationships among nectar sugar components (e.g., sucrose, glucose, fructose) across species. The strength and direction of associations were visualized using scatterplots with regression lines and R^2^ values. Differences in sugar content among species were further evaluated using the Kruskal–Wallis test followed by Dunnett’s multiple comparison test (α = 0.05).

### 2.5. Amino Acid Content and Composition

The collected floral nectar samples were analyzed for amino acids using *O*-phthalaldehyde (OPA) and fluorenylmethyl chloroformate (FMOC) derivatization. The samples were sequentially mixed with borate buffer, OPA/mercaptopropionic acid, and FMOC reagent and then analyzed using an HPLC 1200 series system (Agilent Technologies, Santa Clara, CA, USA). The mobile phase consisted of two solutions: Solution A, containing 10 mM Na_2_HPO_4_ and 10 mM Na_2_B_4_O_7_·10H_2_O at pH 8.2, and Solution B, a mixture of water, acetonitrile, and methanol at a 10:45:45 ratio. The gradient conditions were set to start at 100:0 (*v*/*v*, %) of Solution A to Solution B from 26 to 28 min, changing to 0:100 from 28 to 30.5 min, and then reverting to 100:0 after 30.5 min. The flow rate was maintained at 1.5 mL/min with an injection volume of 0.5 μL. The INNO C-18 column (150 mm × 4.6 mm, 5 μm; Youngjin Biochrom Co., Ltd., Seongnam, Republic of Korea) was maintained at 40 °C. Identification and quantification were conducted by comparing the retention times and peak areas with those of standard amino acids (Sigma-Aldrich, USA), including all essential amino acids. Calibration curves were constructed using serial dilutions (1, 5, 10, 50, and 100 μM), with linearity (R^2^ > 0.995) verified for each amino acid. Results were expressed in mg/L. Amino acid peaks were validated by dual detection: UV (338 nm) and fluorescence (OPA: Ex 340/Em 450 nm, FMOC: Ex 266/Em 305 nm).

Amino acids detected in floral nectar were categorized into three functional groups based on biosynthetic role and physiological relevance: essential amino acids (EAAs), non-essential amino acids (NEAAs), and non-protein amino acids (NPAAs) [55,56].

### 2.6. Floral Nectar Sugar and Amino Acid Production

We estimated the potential production using the floral nectar volume, sugar and amino acid content per unit volume, and the number of flowers per tree and per hectare. The sugar and amino acid production per flower were calculated by multiplying the nectar volume per flower by the respective content per unit volume. Finally, the floral nectar and amino acid yield per hectare were determined using the number of flowers per hectare. In this study, the term ‘production’ refers to the estimated total yield of nectar-derived sugars and amino acids per tree, rather than the physiological synthesis rate within floral tissues.

Sugar yield (kg/ha) = sugar production per tree (g/tree) ^a^ × tree density (tree/ha) ^b^ × 0.001 (for unit conversion: g to kg)

Amino acid yield (g/ha) = amino acid production per tree (mg/tree) ^c^ × tree density (tree/ha) × 0.001 (for unit conversion: mg to g)

^a^ Sugar production per tree (g/tree) = nectar volume per flower (μL/flower) × free sugar content (μg/μL) × number of flower per tree (ea/tree) × 0.00001 (for unit conversion: μg to g)

^b^ Tree density (tree/ha) = 10,000 m^2^/crown width^2 (m^2^)

^c^ Amino acid production per flower (mg/flower) = nectar volume per flower (μL/flower) × free amino acid content (mg/L) × number of flower per tree (ea/tree) × 0.00001 (for unit conversion: μL to L)

### 2.7. Statistics

All statistical analyses were conducted using tree-level mean values in JMP version 18.2.0 (SAS Institute Inc., Cary, NC, USA). Prior to analysis, all datasets were tested for normality and homogeneity of variance using the Shapiro–Wilk and Levene’s tests, respectively. Given the observational nature of the study and moderate sample sizes, we adopted non-parametric methods that are robust to violations of these assumptions.

Interspecific differences in nectar volume, free sugar content, and amino acid composition were evaluated using the Kruskal–Wallis test. When significant differences were identified, Dunnett’s multiple comparison test was applied for post-hoc pairwise comparisons. This analytical framework was selected to enable statistically valid inference without relying on strict distributional assumptions.

## 3. Results

### 3.1. Flowering Phenology and Abundance

The blooming periods and growth characteristics of the eight trees were recorded (Table 1). *S. commixta* and *W. floribunda* began blooming in late April, with flowering periods of 11 and 15 days, respectively. *S. japonicus* and *A. turbinata* started blooming in early May, each with a flowering period of 13 and 15 days, respectively. *C. kousa* bloomed for approximately a month, from mid-May to early July. *T. amurensis* and *K. paniculata* began blooming in mid- to late June, with flowering periods of 14–16 days. The latest blooming tree species was *H. miconioides*, which flowered for 22 days from 24 August to 14 September.

The tree height and crown width were in the range of 2.1–15.7 m and 1.2–11.4 m, respectively. Based on the crown width, the number of trees per hectare (tree density) was calculated, with the smallest crown width of *H. miconioides* (1.2 m) having 7838 trees and the largest crown width of *A. turbinate* (11.4 m) having 77 trees. *W. floribunda*, a vine plant covering a metal frame, did not spread beyond the width of the frame (refer to Figure 1). Therefore, the crown area was determined to be 33 m^2^ (3 m × 11 m). The canopy width was used to quantify the flowering abundance per unit area for each tree.

We surveyed the blooming quantity per tree and multiplied it by the number of trees per hectare, calculated based on canopy width, to estimate the number of flowers per hectare (Table 2). Although *A. turbinata* had the highest number of flowers per tree, *T. amurensis* had the highest number of flowers per hectare. Similarly, while *W. floribunda* produced 3.5 times more flowers per tree than *S. commixta*, their numbers of flowers per hectare were comparable. In contrast, *H. miconioides* produced only 1.9 times more flowers per tree than *C. kousa*, but the difference in the number of flowers per hectare was greater, with *H. miconioides* having 6.2 times more.

### 3.2. Floral Nectar Volume

**Figure 2:**
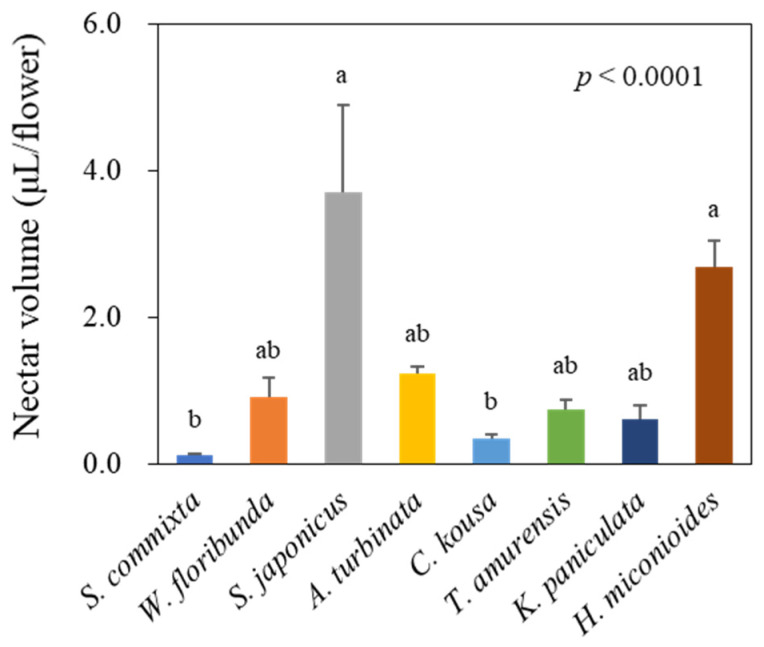
Nectar volume per flower of eight urban tree species. Data represent mean ± standard deviation. Letters above bars indicate statistically significant differences between species (Kruskal–Wallis test followed by Dunnett’s multiple comparison test, *p* < 0.05).

Significant differences in nectar volume per flower were observed among the eight tree species (Figure 2, *p* < 0.0001). *S. japonicus* had the highest nectar volume per flower at 3.71 ± 1.18 μL, followed by *H. miconioides* at 2.67 ± 0.38 μL, *A. turbinata* at 1.23 ± 0.10 μL, *W. floribunda* at 0.90 ± 0.26 μL, *T. amurensis* at 0.72 ± 0.12 μL, *K. paniculata* at 0.61 ± 0.18 μL, *C. kousa* at 0.34 ± 0.06 μL, and *S. commixta* at 0.11 ± 0.03 μL.

### 3.3. Sugar Content and Compositon in Floral Nectar

**Figure 3:**
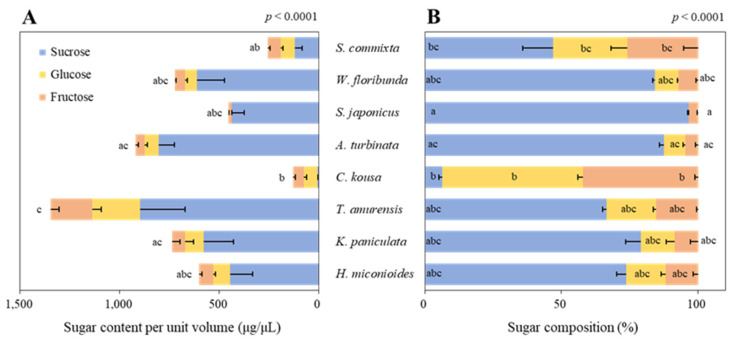
Sugar content per unit volume (**A**) and composition (**B**) of eight urban tree species. Data were analyzed using the Kruskal–Wallis test with post-hoc pairwise comparisons performed using Dunnett’s multiple comparison test at the 5% level. Different letters before (**A**) and within (**B**) each bar indicate statistical differences.

Significant differences in the sugar content per unit volume were observed among the tree species (Figure 3A, *p* < 0.0001). *T. amurensis* had the highest free sugar content per unit volume at 1345.8 ± 313.7 μg/μL, whereas *C. kousa* had the lowest at 128.2 ± 26.2 μg/μL—a difference of > 10-fold. The second-highest free sugar content per unit volume was found in *A. turbinata* (919.5 ± 107.8 μg/μL), followed by *K. paniculata* (735.3 ± 183.8 μg/μL), *W. floribunda* (723.8 ± 154.9 μg/μL), *H. miconioides* (600.8 ± 119.9 μg/μL), *S. japonicus* (453.6 ± 64.5 μg/μL), and *S. commixta* (254.5 ± 20.0 μg/μL).

The sugar composition of the floral nectar showed that all species except *C. kousa* were dominated by sucrose, with significant differences among species (Figure 3B, *p* < 0.0001). In particular, *S. japonicus* contained 96.5% sucrose with no detectable glucose. *W. floribunda* and *A. turbinata* had sucrose compositions of over 80% (84.3 and 87.5%, respectively), while *K. paniculata* and *H. miconioides* had over 70% sucrose (79.0% and 73.6%, respectively). In contrast, *C. kousa* was dominated by glucose and fructose at 51.4% and 42.2%, respectively, compared with only 6.5% sucrose.

**Figure 4:**
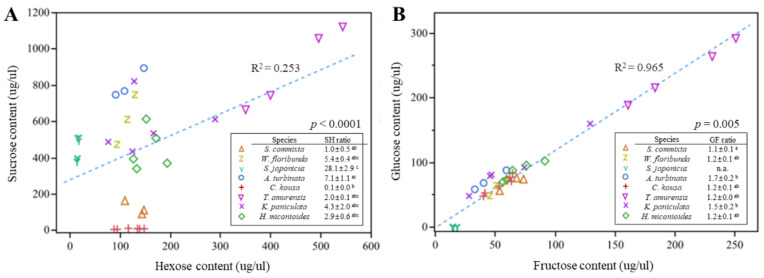
Correlation analysis of sugar content. (**A**) Relationship between sucrose and hexose content in nectar (μg/μL). (**B**) Relationship between glucose and fructose content in nectar (μg/μL). Scatterplots illustrate correlations between sugar components across different tree species, with R^2^ and *p*-values based on linear regression. To compare sugar contents among species, data were analyzed using the Kruskal–Wallis test followed by Dunnett’s multiple comparison test (α = 0.05). Different letters in the legend indicate significant differences among species.

To verify the differences in sugar composition ratios, we analyzed the correlations. The sucrose-to-hexose ratio exhibited different patterns among the tree species (Figure 4A), with significant variability (0.1–28.1, *p* < 0.0001). All tree species, except *C. kousa*, were classified as sucrose-dominant (sucrose 51–100%), whereas *C. kousa* was classified as hexose-dominant (sucrose 0–9%).

A very high positive correlation was found in the ratio of glucose to fructose across all tree species (Figure 4B, R^2^ = 0.965), suggesting that when sucrose is hydrolyzed into glucose and fructose, they are produced at nearly equal concentrations, with glucose being slightly more prevalent than fructose (>1.0). However, variability among the tree species was observed (*p* = 0.0002).

### 3.4. Amino Acid Content in Floral Nectar

**Figure 5:**
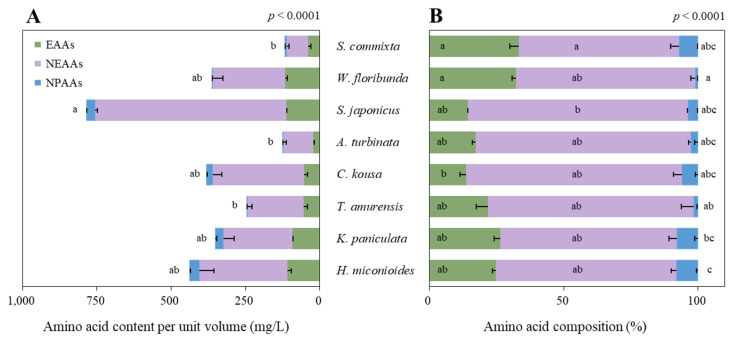
Amino acid content per unit volume (**A**) and amino acid composition (**B**) of the eight urban trees in Korea. Data were analyzed using the Kruskal–Wallis test with post-hoc comparison using Dunnett’s multiple comparison test at the 5% level. Different letters before (**A**) and within (**B**) each bar indicate statistical differences. EAAs, essential amino acids; NEAAs, non-essential amino acids; NPAAs, non-protein amino acids.

Significant differences in the content of amino acids per unit volume were observed among the tree species (Figure 5A, *p* < 0.0001). *S. japonicus* had a significantly higher amino acid content (785.2 ± 11.4 μg/L) compared with those of the other tree species, followed by *H. miconioides* with 439.3 ± 63.7 μg/L. *W. floribunda*, *C. kousa*, and *K. paniculata* had similar amino acid contents of 363.4 ± 43.1, 381.9 ± 31.2, and 351.2 ± 38.3 μg/L, respectively, while *S. commixta* (118.6 ± 18.6 μg/L) and *A. turbinata* (125.0 ± 11.5 μg/L) had the lowest amino acid contents per unit volume.

The analysis of the amino acid composition in the floral nectar, categorized into essential, non-essential, and non-protein amino acids, also revealed significant differences among the species (Figure 5B, *p* < 0.0001). Generally, all species had non-essential amino acids (59.7–80.3%) as the largest proportion, followed by essential amino acids (13.6–33.3%) and non-protein amino acids (1.0–8.1%). *S. commixta* and *W. floribunda* had higher proportions of essential amino acids than those of the other tree species, whereas *K. paniculata* and *H. miconioides* had relatively higher proportions of non-protein amino acids.

**Figure 6:**
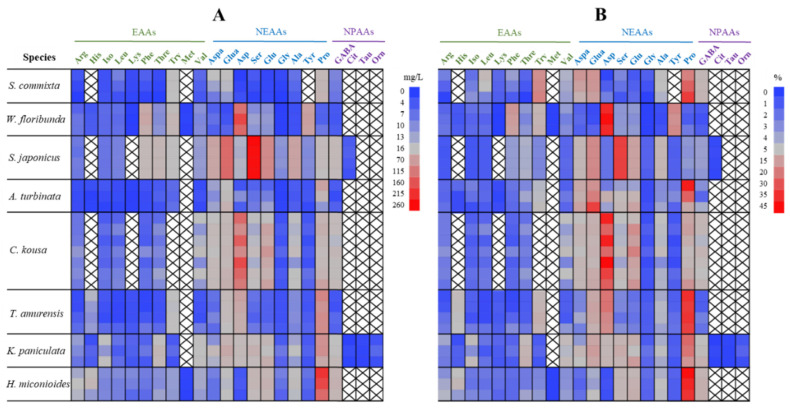
Comparison of amino acid concentration (mg/L) (**A**) and composition (%) (**B**) in floral nectar of eight street tree species in Korea. Each row represents an individual tree. Amino acids are functionally grouped into essential amino acids (EAAs), non-essential amino acids (NEAAs), and non-protein amino acids (NPAAs). Arg, arginine; His, histidine; Iso, isoleucine; Leu, leucine; Lys, lysine; Phe, phenylalanine; Thre, threonine; Try, tryptophane; Val, valine; Aspa, aspartic acid; Glua, glutamic acid; Asp, asparagine; Ser, serine; Glu, glutamine; Gly, glycine; Ala, alanine; Tyr, tyrosine; Pro, proline; GABA, γ-Aminobutyric Acid; Cit, citrulline; Tau, taurine; Orn, ornithine.

The floral nectar of the eight trees contained 16–22 amino acid types (Figure 6A). Among the 10 essential amino acids, arginine, isoleucine, leucine, phenylalanine, threonine, and valine were present in all the tree species, whereas methionine was found only in *W. floribunda* and *H. miconioides*. *W. floribunda* and *H. miconioides* contained all ten essential amino acids; *A. turbinata*, *T. amurensis*, and *K. paniculata* contained nine; *S. commixta* contained eight; and *S. japonicus* and *C. kousa* contained seven and six amino acids, respectively. All the nine non-essential amino acids were found in all the tree species, except for tyrosine, which was absent in *S. commixta*. Among the non-protein amino acids, GABA was found in all the tree species. In contrast, citrulline was found only in *S. japonicus* and *K. paniculata*, and taurine and ornithine were found only in *K. paniculata*.

Among the 15–23 amino acids found in each species, the dominant amino acids (comprising more than 10% of the total) were as follows (Figure 6B, shown in light red to red): proline (26.0%), tryptophan (16.6%), glutamic acid (13.2%), and aspartic acid (10.8%) in *S. commixta*; asparagine (39.5%), tyrosine (16.2%), and phenylalanine (12.6%) in *W. floribunda*; serine (32.6%) and glutamic acid (16.8%) in *S. japonicus*; proline (25.3%) and glutamic acid (22.0%) in *A. turbinata*; asparagine (32.9%) and glutamine (12.5%) in *C. kousa*; proline (34.4%) and asparagine (18.7%) in *T. amurensis*; proline (19.6%) and glutamic acid (10.9%) in *K. paniculata*; and proline (39.0%) in *H. miconioides*.

### 3.5. Nectar Sugar Production

**Figure 7:**
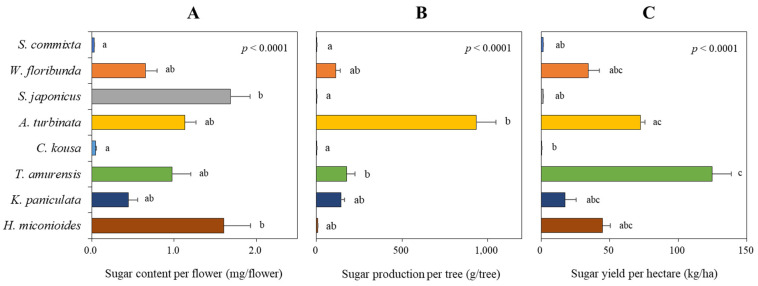
Assessment of sugar content per flower (**A**), sugar production per tree (**B**), and sugar yield per hectare (**C**) of eight tree species. Data were analyzed using the Kruskal–Wallis test with post-hoc comparison using Dunnett’s multiple comparison test at the 5% level. Different letters after each bar indicate significant differences.

The sugar content per flower, calculated by multiplying the nectar volume per flower by the sugar content per unit volume, showed significant variability among the tree species (Figure 7A, *p* < 0.0001). *S. japonicus* (1.68 ± 0.24 mg/flower) and *H. miconioides* (1.61 ± 0.32 mg/flower) had higher values than the other species. They were followed by *A. turbinata* (1.13 ± 0.13 mg/flower), *T. amurensis* (0.97 ± 0.23 mg/flower), *W. floribunda* (0.65 ± 0.14 mg/flower), *K. paniculata* (0.45 ± 0.11 mg/flower), *C. kousa* (0.04 ± 0.01 mg/flower), and *S. commixta* (0.03 ± 0.00 mg/flower).

The sugar production per tree, calculated by multiplying the sugar content per flower with the total number of flowers per tree, significantly changed the ranking among the tree species (Figure 7B, *p* < 0.0001). *A. turbinata* showed the highest production (932.7 ± 117.7 g/tree), followed by *T. amurensis* (177.4 ± 48.9 g/tree), *K. paniculata* (142.2 ± 22.8 g/tree), and *W. floribunda* (113.3 ± 26.6 g/tree). *H. miconioides* (5.9 ± 2.1 g/tree), *S. commixta* (1.4 ± 0.6 g/tree), *S. japonicus* (0.9 ± 0.2 g/tree), and *C. kousa* (0.1 ± 0.0 g/tree) showed relatively low production.

The sugar yield per hectare (Figure 7C), based on the number of flowers per hectare, was the highest in *T. amurensis* (87.6 ± 24.1 kg/ha), followed by *A. turbinata* (71.8 ± 9.1 kg/ha). *H. miconioides* (41.1 ± 14.8 kg/ha), *W. floribunda* (34.3 ± 8.1 kg/ha), and *K. paniculata* (24.0 ± 3.8 kg/ha) showed relatively good floral nectar production, whereas *S. commixta* (1.4±0.6 kg/ha), *S. japonicus* (1.4 ± 0.3 kg/ha), and *C. kousa* (0.2 ± 0.1 kg/ha) exhibited very low performance (*p* < 0.0001).

### 3.6. Nectar Amino Acid Production

**Figure 8:**
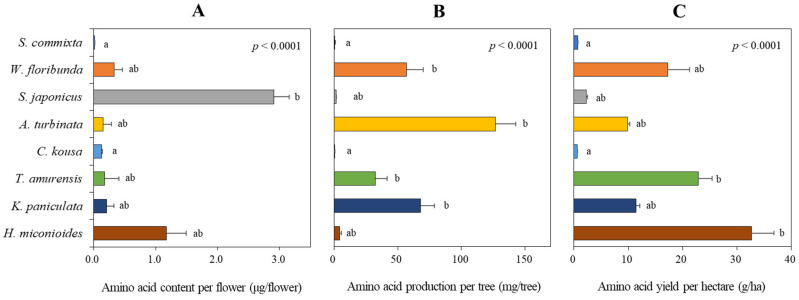
Assessment of amino acid content per flower (**A**), amino acid production per tree (**B**), and amino acid yield per hectare (**C**) of eight tree species. Data represent the mean ± standard deviation. Data were analyzed using the Kruskal–Wallis test with post-hoc comparison using Dunnett’s multiple comparison test at the 5% level. Different letters after each bar indicate significant differences.

*S. japonicus* had the highest amino acid content per flower at 2.91 ± 0.04 μg/flower, followed by *H. miconioides* at 1.17 ± 0.17 μg/flower. The other six species had amino acid production per flower ranging from 0.13 to 0.33 μg/flower, with *S. commixta* having the lowest content (Figure 8A).

Based on the number of flowers per tree (Figure 8B), *A. turbinata* had the highest amino acid production per tree at 126.8 ± 16.0 mg/tree, followed by *K. paniculata* (67.9 ± 10.9 mg/tree), *W. floribunda* (56.9 ± 13.4 mg/tree), and *T. amurensis* (32.5 ± 9.0 mg/tree), all showing significant production levels. In contrast, the other four species (*S. commixta*, *S. japonicus*, *C. kousa*, and *H. miconioides*) had very low productivity, ranging from 0.6 to 4.3 mg/tree.

In terms of amino acid yield per hectare (Figure 8C), *H. miconioides* had the highest at 30.1 g/ha, followed by *W. floribunda* (17.2 ± 4.1 g/ha), *T. amurensis* (16.1 ± 4.4 g/ha), *K. paniculata* (11.5 ± 1.8 g/ha), and *A. turbinata* (9.8 ± 1.2 g/ha). The other three species (*S. commixta*, *S. japonicus*, and *C. kousa*) had very low yields, ranging from 0.6 to 2.4 g/ha.

## 4. Discussion

### 4.1. Standardized Approach to Assessing Floral Resource Yield

Quantifying floral resources, particularly sugar and amino acid production, is crucial for evaluating the ecological value of plant species to pollinators [28,76]. Significant variability has been reported not only between species [32,33,77,78,79] but also within varieties of the same species [34,75,80]. Particularly in woody species, such assessments are complicated by natural variation in age, canopy size, and flower density [67,68]. This underscores the need for applying consistent and standardized measurement criteria across studies [69].

In our study, floral resource productivity was assessed at multiple scales—per flower, per tree, and per hectare—to facilitate equitable interspecific comparisons. This approach revealed substantial interspecific variation in both sugar and amino acid outputs. For instance, *T. amurensis*, despite ranking fifth in nectar secretion per flower (Figure 2), produced the highest sugar yield per hectare (Figure 7C) due to its high sugar concentration (Figure 3A) and abundant flower density (Table 2). Conversely, *S. japonicus*, with its high nectar volume per flower (Figure 2), was among the lowest in hectare-level productivity owing to limited flower abundance (Figure 7C and Figure 8C).

Likewise, *H. miconioides* exhibited high sugar and amino acid content per flower but ranked lower in per-tree productivity due to its small canopy area (1.3 m^2^). Yet, when scaled to potential planting density per hectare, it ranked third in sugar and first in amino acid yield per hectare (Figure 7 and Figure 8), highlighting how scaling factors can shift interpretations. Similarly, *A. turbinata*, despite low nectar volume and amino acid content per flower, achieved high per-tree amino acid production due to prolific flowering. However, its hectare-level output was moderate (fifth in amino acid yield per hectare), suggesting that tree size and structure must be carefully accounted for during resource assessments.

These findings affirm that isolated trait measurements, such as nectar volume per flower, can misrepresent a species’ overall ecological contribution. Therefore, multi-scale standardized assessments remain essential for evaluating floral resource value in urban planting strategies.

### 4.2. Species-Level Differences in Nutritional Yield and Urban Planting Implications

The survival and development of pollinators depend on the diversity and abundance of floral resources [19,81]. Therefore, the selection of urban tree species must consider not only aesthetic value but also their nutritional contributions in terms of nectar and amino acid production [38,82]. Declines in pollinator populations have been associated with floral resource scarcity and dietary homogenization, which create seasonal ‘food gaps’ due to variations in flowering phenology [8,39,40,83]. Managing these gaps requires detailed knowledge of flowering periods and nutrient supply across species [35].

Our study quantified sugar and amino acid production in eight urban tree species in South Korea. We found that *T. amurensis* demonstrated the highest sugar productivity (87.6 kg/ha), followed by *A. turbinata* (71.8 kg/ha), *H. miconioides* (41.1 kg/ha), and *W. floribunda* (34.3 kg/ha) (Figure 7C). These findings are significant for urban beekeeping development [84], as they suggest species that provide substantial nectar resources.

In terms of amino acid productivity, *H. miconioides* ranked first (30.1 g/ha), followed by *W. floribunda* (17.2 g/ha), *T. amurensis* (16.6 g/ha), and *K. paniculata* (11.5 g/ha) (Figure 8C). Interestingly, rankings for sugar and amino acid productivity were inconsistent across species (Figure 7C and Figure 8C). For instance, *T. amurensis*, which had the highest sugar production, ranked third in amino acid production, while *A. turbinata* was second in sugar but fifth in amino acid yield. Conversely, *S. commixta*, *S. japonicus*, and *C. kousa* exhibited low productivity for both sugars and amino acids, despite being listed as nectar plants under the Korean Beekeeping Industry Promotion and Support Act. This discrepancy underscores the necessity of empirical validation over generalized assumptions that may have been institutionalized in policy [18,28].

To maximize pollinator support, urban landscapes should incorporate species with complementary flowering phenology. This ensures continuous food availability across seasons, addressing temporal gaps [26,85,86,87,88]. In addition to trees, herbaceous and vine plants can further enhance floral continuity by blooming during periods when tree species are not flowering, thereby contributing to year-round foraging opportunities for pollinators.

**Figure 9:**
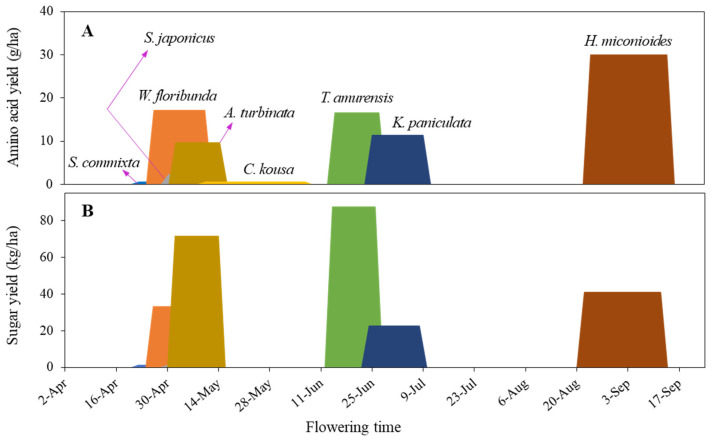
Amino acid yield per hectare (**A**) and sugar yield per hectare (**B**) by flowering time. By continuously accumulating such data, we can identify urban pollinators with nutritionally balanced and continuous food resources year-round.

Additionally, tree selection must balance ecological function with public preferences, ensuring species are pollinator-friendly and appreciated by urban residents [89]. Such integrated planning forms the basis for multifunctional urban biodiversity strategies [27,90]. We propose the continuous accumulation of data, such as that shown in Figure 9, which displays blooming periods, sugar, and amino acid productivity, to enhance and manage sustainable habitats and food resources for pollinators in urban settings. This approach will assist urban landscape design by ensuring the continuity of flower resource availability throughout the year and the addition of suitable species to fill food gaps.

### 4.3. Floral Nectar Composition and Its Functional Implications for Pollinators

With the increasing need for pollinator conservation, it has become essential to evaluate not only the availability of food resources for ornamental plants but also the quality of floral nectar, which influences the growth, colony development, and immune systems of pollinators [91,92]. Specifically, the quality and quantity of sugars are key factors affecting the frequency of visits by pollinators [61,62,93]. Among the most prevalent pollinator groups in urban areas, bees [25] prefer floral nectar that combines sucrose, glucose, and fructose [94], selecting floral nectar with higher concentrations of these compounds to optimize energy intake. According to our findings, the sugar composition of eight studied tree species revealed that *T. amurensis*, *A. turbinata*, *K. paniculata*, and *W. floribunda* exhibit a high sugar content, exceeding 700 μg/μL per unit volume (Figure 3A), indicating a favorable composition for bees. Conversely, butterflies, a pollinator group significantly affected by urbanization [95], prefer low-viscosity floral nectar owing to ease of consumption with their long proboscis [96]. The tree species *S. japonicus*, *S. commixta*, and *C. kousa* align with this preference (Figure 3A).

The quality of floral nectar is influenced not only by sugar content but also by amino acid composition [48,49], which can affect pollinator foraging behavior and floral preference [50,97]. While pollen is generally considered the primary source of amino acids for pollinators [51], nectar-borne amino acids may still play ecologically significant roles—especially for adult insects such as honeybees and butterflies that rely heavily on nectar as their main food source. The response to nectar amino acids varies across pollinator taxa: butterflies tend to prefer higher amino acid concentrations, whereas bees often forage from flowers with relatively lower amino acid ratios [98,99]. Although the functional significance of nectar amino acids remains under debate [56], their presence has been associated with changes in pollinator foraging behavior and preference across multiple insect groups [52,53,54].

Our study not only highlights the significant differences in amino acid productivity among the eight species but also emphasizes the compositional differences between them. The variation in amino acid composition among species that bloom at different times can potentially provide pollinators with more nutritionally balanced food resources. For instance, in this study, the floral nectar of *S. commixta* lacked histidine, methionine, and tyrosine; however, these compounds were present in *W. floribunda*, which bloomed at a similar time. Similarly, *S. japonicus* floral nectar lacked histidine, lysine, and methionine, but these were present in the floral nectar of *A. turbinata*, which bloomed almost simultaneously. Conversely, *S. japonicus* had elevated levels of phenylalanine, threonine, tryptophan, aspartic acid, glutamic acid, serine, and glutamine, which were relatively low in *A. turbinata*. Considering the complementary relationship between *T. amurensis* and *K. paniculata*, which bloom sequentially, *T. amurensis* had higher levels of histidine, tryptophan, and asparagine, whereas *K. paniculata* had higher levels of isoleucine, threonine, aspartic acid, and serine (Figure 5A). This confirmed that the qualitative and quantitative composition of floral nectar amino acids varies between species [63,100]. The functions of each amino acid are documented in the article of Kostryco and Chwil (2022) [101].

Recent studies have highlighted the importance of non-protein amino acids (NPAA) in floral nectar. The functional significance of NPAA [56], their biological roles [102], and their relationship with different pollinators [103] are still ongoing research. Carlesso et al. (2021) [104] proposed that NPAA in floral nectar might be a cooperative strategy that enhances the transfer of pollen between conspecific plants by encouraging pollinators to learn and recognize the traits of the associated flowers. Additionally, Nepi (2014) [97] noted that NPAA in floral nectar could potentially influence floral nectar-mediated plant-animal interactions and play a role in protecting floral nectar from microbial invasion. Our study found that the NPAA concentrations in the floral nectar of the eight tree species ranged from 1.0% to 8.1% of total amino acid contents across species, with significant variations (Figure 6B). GABA was consistently identified as the most abundant NPAA (Figure 6). GABA is a prominent NPAA in floral nectar [19,105] and the leading inhibitory neurotransmitter in the insect brain [106]. It plays a crucial role in olfactory processing and learning [107,108] and enhances honeybee memory capabilities [104]. Furthermore, taurine, which was found only in *K. paniculata*, is recognized as an important neuromodulator in the insect brain, alongside GABA and *β*-alanine, which can interact with the neural activity of nectar foragers shortly after ingestion [109].

### 4.4. Study Limitations

While our study focused on nectar composition and quantifiable chemical rewards, it is important to recognize that non-chemical floral traits also play a pivotal role in pollinator attraction and resource utilization. Features such as floral morphology, corolla depth, flower height from the ground, and exposure to wind or sunlight influence the accessibility and stability of nectar resources, particularly in urban environments with fragmented microhabitats [110,111]. For example, short-tongued bees may be excluded from deep, tubular flowers, while long-proboscid Lepidoptera and specialized flies may exploit these efficiently [112]. Such structural constraints also affect the foraging efficiency and species-specific floral constancy of pollinators.

In addition, although our primary focus was on honeybees and butterflies, which are dominant and ecologically relevant in East Asian urban flora, it is worth noting that a wide range of animal pollinators exist globally. These include flies, beetles, moths, birds, bats, and even some non-flying mammals, which contribute to pollination in tropical and temperate ecosystems [1,113]. Although such vertebrate pollinators are rarely documented in Korean urban landscapes, this broader perspective underscores the value of incorporating diverse pollination strategies in landscape planning.

We recommend that future studies combine both chemical and structural floral traits to provide a more holistic evaluation of plant species for pollinator conservation. Such integrated assessments will better inform tree selection in multifunctional urban greening projects.

We also acknowledge that data were collected during a single flowering season (2022). Floral resource availability—including nectar volume, sugar concentration, and flowering phenology—can vary considerably across years in response to environmental conditions such as temperature, rainfall, and humidity [40,68]. This temporal variability can affect the continuity of floral resources and may result in seasonal food gaps for pollinators. To address this, future studies should adopt long-term, multi-year monitoring frameworks that integrate both chemical and structural floral traits to provide a more holistic and resilient basis for pollinator-friendly urban greening strategies.

## 5. Conclusions

We quantitatively evaluated the availability of floral resources (total sugar and amino acid production) from eight tree species recommended for planting in urban green spaces in South Korea. Although these species are recognized as commonly recommended floral nectar plants in the country, information provided by non-experts should be approached with caution. Our findings revealed that sugar content varied by up to 438-fold, while amino acid production differed by up to 50-fold among species. Additionally, we found that tree species with high total sugar production did not consistently align with those that had high amino acid production. Moreover, our results indicate that the selection of pollinator-friendly plant species should be based on a thorough survey and analysis of various factors, including floral nectar volume, concentration per unit volume, and standardized flower numbers per tree and per hectare. In addition to these quantitative measures, future tree selection should also consider ecological traits such as flowering phenology, floral morphology, accessibility to diverse pollinator taxa, and native species status. These dimensions contribute to more resilient, multifunctional, and pollinator-friendly urban ecosystems.

## Figures and Tables

**Table 1:** Growth and flower characteristics of eight tree species.

Species	n	Flowering Period	Height (m)	Canopy Area (m^2^)	Calculated Tree Density (tree/ha)
*S. commixta*	8	Apr 23–May 3	3.3 ± 0.5	9.7 ± 0.1	1043 ± 106
*W. floribunda*	3	Apr 27–May 11	3.0	33 *	303
*S. japonicus*	4	May 1–May 15	3.3 ± 0.2	7.1 ± 0.6	1417 ± 131
*A. turbinata*	3	May 3–May 15	15.7 ± 0.8	129.8 ± 8.8	77 ± 5
*C. kousa*	6	May 12–Jun 7	3.9 ± 1.7	4.5 ± 0.9	2287 ± 430
*T. amurensis*	4	Jun 16–Jun 28	4.8 ± 1.4	14.3 ± 1.5	703 ± 79
*K. paniculata*	5	Jun 24–Jul 9	5.6 ± 0.9	59.3 ± 3.3	169 ± 9
*H. miconioides*	5	Aug 24–Sep 14	2.1 ± 0.3	1.3 ± 0.2	7552 ± 952

* *W. floribunda* survey data describes the vine plant covering the roof of an iron frame, with the length and width indicated (refer to Figure 1).

**Table 2:** Flowering abundance from eight tree species.

Species	Number of Flowers per Tree (Thous.)		Number of Flowers per Hectare (Thous.)	
	Mean ± SD	Min.–Max.	Mean ± SD	Min.–Max.
*S. commixta*	49.5 ± 20.7	21.6–82.1	51,629 ± 5240	42,825–60,949
*W. floribunda*	174.0 ± 40.9	134.6–216.2	52,723 ± 12,393	40,785–65,526
*S. japonicus*	0.6 ± 0.0	0.4–0.7	794 ± 73	741–896
*A. turbinata*	824.7 ± 104.1	720.6–928.8	63,861 ± 3093	60,782–66,967
*C. kousa*	2.0 ± 0.7	1.1–2.6	4522 ± 850	3581–5477
*T. amurensis*	182.3 ± 50.0	100.4–279.6	128,119 ± 14,313	119,855–144,646
*K. paniculata*	318.3 ± 50.9	267.4–369.2	53,744 ± 2948	51,659–55,828
*H. miconioides*	3.7 ± 1.3	2.0–6.2	27,840 ± 3511	21,813–30,466

## Data Availability

Data are contained within the article.
